# Epidemiology of infective endocarditis in French intensive care units over the 1997–2014 period—from CUB-Réa Network

**DOI:** 10.1186/s13054-019-2387-8

**Published:** 2019-04-25

**Authors:** Jérémie Joffre, Guillaume Dumas, Philippe Aegerter, Vincent Dubée, Naike Bigé, Gabriel Preda, Jean-Luc Baudel, Eric Maury, Bertrand Guidet, Hafid Ait-Oufella, on behalf of the CUB-Réa Network, P. Trouiller, J.-P. Bedos, A. Vieillard-Baron, Y. Cohen, C. Richard, J. F. Timsit, G. Chevrel, J.-P. Mira, D. Da Silva, J.-L. Diehl, P. Ho, A. Mekontso-Dessap, F. Blot, G. Dhonneur, D. Dreyfuss, B. Megarbane, Goldgran Toledano Dany, V. Das, D. Samuel, A. Demoule, A. Combes, F. Bolgert, H. Outin, F. Santoli, D. Annane, B. Guidet, C. Bruel, E. Azoulay, A. Mebazaa, M. Fartoukh, F. Bonnet, H. Mentec

**Affiliations:** 1 Medical Intensive Care Unit, Hôpital Saint-Antoine, Assistance Publique-Hôpitaux de Paris, 75571 Paris CEDEX 12, France; 2 INSERM U970, Cardiovascular Research Center, Hôpital Européen Georges Pompidou, Assistance Publique-Hôpitaux de Paris, 75015 Paris, France; 3 INSERM UMR S1168, Hôpital Ambroise Paré UFR Médecine Paris Ile de France-Ouest, 92100 Boulogne, France; 4 Infectious and Tropical Disease Department, Hôpital universitaire d’Angers, 49100 Angers, France; 5 Sorbonne Universités, UPMC University Paris 06, INSERM, UMRS 1136, Institut Pierre Louis d’Epidémiologie et de Santé Publique, Paris, France; 6 Hôpital Saint-Antoine, Assistance Publique-Hôpitaux de Paris, 184 Rue du Faubourg Saint-Antoine, 75571 Paris CEDEX 12, France

**Keywords:** Infective endocarditis, Critical care, Epidemiology, Outcome, Surgery

## Abstract

**Background:**

Few studies focus only on severe forms of infective endocarditis, for which organ failure requires admission to an intensive care unit (ICU). This study aimed to describe demographical, comorbidities, organ failure, and pathogen-related characteristics in a population of critically ill patients admitted to ICU for infective endocarditis and to identify risk factors of in-ICU mortality.

**Methods:**

Retrospective observational multicenter (*N* = 34) study of the CUB-Rea register, based on ICD-10 coding rules, between 1997 and 2014 in France including ICU patients managed for infective endocarditis. In-ICU mortality associated factors were assessed by multivariate logistic regression including an interrupted time analysis of three periods (1997–2003, 2004–2009, and 2010–2014).

**Results:**

Four thousand four hundred five patients admitted in ICU for infective endocarditis were included. We observed an increase in endocarditis prevalence, as well as an increase in organ failure severity over the three periods. In addition, valve surgery was more frequently performed (27%, 31%, and 42%, *P* < 0.0001) while in-ICU mortality significantly decreased (28%, 29%, and 23%, *P* < 0.001). Since 2010, a significant increase in the trends’ slope of incidence for *Streptococcus* sp. and *Staphylococcus* sp. was observed with no change concerning intracellular bacteria, *Enterococcus* sp. or *Candida* sp. slope trends. In multivariate analysis, age, SAPS2, organ failure, stroke, and *Staphylococcus* sp. were associated with ICU mortality. Conversely, surgery, intracardiac devices, male gender, and *Streptococcus* sp.-related infective endocarditis were associated with a better outcome.

**Conclusions:**

Our study reveals a shifting landscape of infective endocarditis epidemiology in French ICUs, characterized by reduced in-ICU mortality despite higher severity, more surgery, and substantial changes in microbial epidemiology.

**Electronic supplementary material:**

The online version of this article (10.1186/s13054-019-2387-8) contains supplementary material, which is available to authorized users.

## Background

Infective endocarditis is an uncommon condition responsible for high morbidity and mortality [1]. Epidemiologists estimate that the number of new infective endocarditis cases will reach 2000–2500/year in France in the next years [2, 3]. Despite some discrepancies between studies, infective endocarditis incidence seems to increase over time in the USA [4] and Europe [5, 6]. Besides, significant changes have been reported in infective endocarditis epidemiology concerning pathogens and patients’ characteristics [3]. Most of these data were obtained from patients managed in internal medicine and cardiology departments, but characteristics of the subgroup of critically ill patients with infective endocarditis and infective endocarditis organ failure are not well known [7]. Our study aimed to describe the demographical, clinical, and microbial patterns of critically ill patients admitted in intensive care unit (ICU) for infective endocarditis and to investigate in-ICU mortality-related factors.

## Methods

### The database

The database of the *Collège des Utilisateurs des Bases des données en Réanimation* (CUB-Réa) included prospectively collected data from 34 (22 academics) ICUs in Paris and its suburb. The database [8] has been fully described elsewhere [9–11]. Briefly, standardized information, both administrative and medical, are collected locally according to the clinical cataloging system ICD-10 (International Classification of Diseases, Tenth Revision) coding rules. Data are gathered prospectively for all patients hospitalized in the ICUs and are transmitted anonymously to the administrative center to be recorded in a relational database. All ICU stays are referred to the hospital diagnosis-related group. Each hospital controls the completeness of coding, so that there are no missing patients or information regarding ICU stays’ characteristics. Coding methods are regularly harmonized among the ICUs. Quality controls confirmed the overall reliability of the data, as previously shown [12]. Data were extracted from 1997 to 2014, corresponding to more than 340,000 admissions to the ICUs participating in the database during the entire period. The list of participating centers is provided in the “Appendix” section.

### Patients’ selection and data collection

For this study, all ICU stays with a primary or secondary diagnosis of infective endocarditis (ICD-10 code I.330) were included and analyzed. The following variables were extracted: demographic characteristics, severity-of-illness assessed by the Simplified Acute Physiology Score 2 (SAPS2) [13], comorbidities, organ supports, pathogen(s) or pathogen family most likely involved according to ICD-10 limitations, infective endocarditis complications, surgery, length of stay in ICU and in-hospital, and vital status at ICU discharge. To avoid duplicates, based on the dates of birth and dates of stays, we identified inter-center transfers and readmissions. Also, the stays identified as transfers or early readmissions (< 1 month after the ICU discharge) were merged into one single stay. Readmissions for endocarditis beyond 1 month of the resuscitation outing were considered recurrent endocarditis, so a new case.

#### Statistical analysis

Results are reported as means (± SD) or medians (IQR) for continuous variables and as percentages for qualitative variables. To figure out associations between patient patterns and ICU outcome, we first performed univariate prognosis analyses based on Wilcoxon Rank sum test or Kruskal-Wallis test for quantitative data, and for qualitative data, chi-square test or Fisher’s exact test, as appropriate. To identify independent predictors of in-ICU mortality, characteristics available at ICU admission associated with *P* values less than 0.1 by univariate analysis or deemed clinically relevant were included in a multivariable logistic regression model with backward selection. Because missing data were accounting for less than 10% of patients, analyses were performed on complete cases (*n* = 4370). Log-linearity for continuous variables was checked. Goodness of fit of the model was assessed using the Le Cessie-van Houwelingen test and discrimination by the area under the receiver operating characteristic curve. Interaction tests (the Gail and Simon test) were conducted to assess heterogeneity in effects across subsets (surgery vs. no surgery and periods 1994–2003, 2004–2009, and 2010–2014). To investigate a potential center effect, the model was also fitted with centers introduced as clusters and random variables.

All tests were two-sided, and *P* values less than 0.05 were considered statistically significant. Statistics were performed using *R* (https://www.R-project.org/) software, and graphical representations were performed using GraphPad Prism 5.04 (GraphPad Software Inc.®).

## Results

**Fig. 1:**
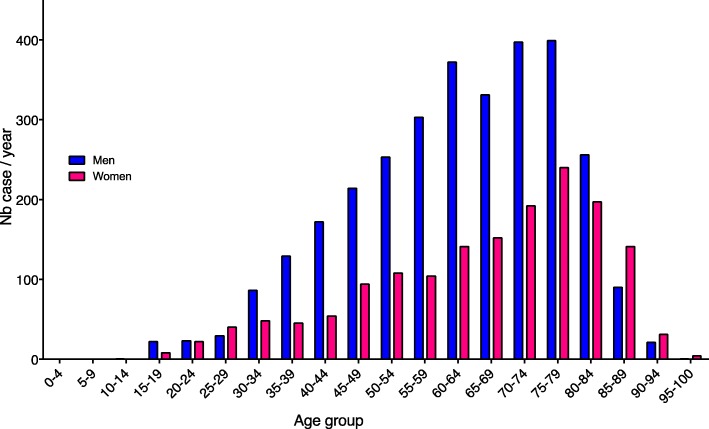
Distribution of infective endocarditis according to gender and age

**Fig. 2:**
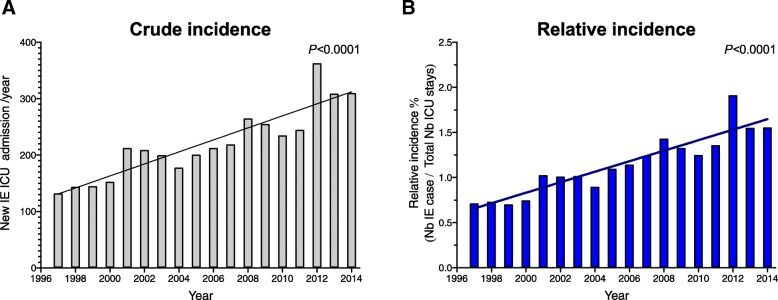
Crude (**a**) and relative (**b**) observed annual incidence of infective endocarditis in ICU over the 1997–2014 period. The shaded regions indicate 95% confidence intervals

In our database, we identified 4757 stays for infective endocarditis over the 18-year period. Among these stays, we identified 352 patients with early readmissions con, and so, ultimately 4405 patients were included in our analysis (Additional file 1: Figure S1). The mean age was 65 ± 16 years with a 2-fold higher incidence in men in the overall cohort (65%) but a reversal of the sex ratio after 80 years old (Fig. 1). Patient’s characteristics are summarized in Table 1a. Among included patients, 12% had prosthetic-valve endocarditis, 4% had cardiac device-related infective endocarditis (implantable pacemaker and/or defibrillator), and 1% had pre-existing congenital heart disease. Overall, 580 patients had diabetes (13.7%), 40 had HIV infection (3.2%), 206 liver cirrhosis (4.7%), and 402 patients (9.1%) had active cancer or hematological malignancies. Over the 18 years, we observed an increase in the number of infective endocarditis patients admitted to ICU (slope 10.7 ± 1.3, *P* < 0.001). Besides, the prevalence of patients with infective endocarditis in ICUs (i.e., adjusted on the number of ICU stays) significantly increased (Fig. 2a and b). Interrupted time analysis did not show a significant rupture in incidence or prevalence trends over the observation period.

**Table 1:** General characteristics of patients included (A), management and outcome features (B). *Abbreviations*: *COPD* chronic obstructive pulmonary disease, *HIV* human immunodeficiency virus, *IV* for intravenous, *SAPS* Simplified Acute Physiology Score, *AV* atrioventricular

A	
Patients characteristics	(*n* = 4405)
Age: mean ± SD	65 ± 16
Gender male: *n* (%)	2866 (65)
SAPS2 (mean ± SD)	46 ± 22
Coexisting condition or risk factors: *n* (%)	
Diabetes	580 (14)
High blood pressure	938 (21)
Cancer and hematological malignancies	402 (9)
COPD and chronic respiratory failure	413 (9)
HIV	140 (3)
IV drug abuse	135 (3)
Dialysis dependent chronic kidney disease	133 (3)
Liver cirrhosis	206 (5)
Intra cardiac material: *n* (%)	595 (14)
Prosthetic valve	527 (12)
Pace maker and/or intra-cardiac defibrillator	160 (4)
Congenital cardiopathy: *n* (%)	62 (1)
Pathogens: *n* (%)	
*Staphylococcus* sp.	1404 (32)
*Streptococcus* sp. (except *S. pneumoniae*)	774 (18)
*Enterococcus* sp.	184 (4)
*Streptococcus pneumoniae*	96 (2)
*Pseudomonas aeruginosa*	124 (3)
Intra cellular	269 (6)
*Candida* sp.	122 (3)
HACEK and Enterobacteriacceae	354 (8)
B	
Patients management and outcomes	(*n* = 4405)
Surgery: *n* (%)	1502 (34)
Acute respiratory failure: *n* (%)	2899 (66)
Mechanical ventilation	
Invasive	2521 (57)
Noninvasive	400 (9)
Invasive ventilation duration: days (median (IQRs))	5 (2–13)
Renal replacement therapy: *n* (%)	1053 (24)
Acute circulatory failure: *n* (%)	2409 (55)
Neurological complication: *n* (%)	780 (18)
Ischemic stroke	459 (10)
Intracranial bleeding	228 (5)
Meningitis	132 (3)
Cerebral abscess	69 (2)
Embolic complications (except neurological): *n* (%)	128 (2.9)
Acute limb ischemia	93 (2.1)
Splenic infarction	20 (0.5)
Kidney infarction	16 (0.4)
Liver infarction	5 (0.1)
Secondary infectious location (except neurological): *n* (%)	246 (5.6)
Septic arthritis	100 (2.3)
Splenic abscess	20 (0.5)
Kidney abscess	23 (0.5)
Liver abscess	17 (0.4)
Psoas abscess	11 (0.2)
Spondylodiscitis	23 (0.5)
Pulmonary abscess	72 (1.6)
High grade AV block	258 (6)
Death in ICU: *n* (%)	1168 (26)
Death in hospital: *n* (%)	1403 (32)
Length of stay in ICU: days (median (IQRs))	6 (3–13)
Length of stay in hospital: days (median (IQRs)	19 (8–38)

### Intervention and outcome

The mean SAPS2 was 46 ± 22, 66% of patients experienced respiratory failure, and most of them required invasive mechanical ventilation (87%). More than 23% of the patients had acute kidney injury requiring renal replacement therapy (RRT), and 53% had septic and/or cardiogenic shock, defined by inotrope and/or vasopressor infusion requirement, extracorporeal life support, and/or intra-aortic balloon pump. During ICU stay, 36% of patients underwent valve cardiac surgery, including valvuloplasty or valve replacement. Endocarditis-related complications have been reported, such as neurological injury (18%) mainly due to ischemic stroke (10%), extra-cerebral embolism (2.9%), secondary septic localization (5.6%), and high-grade atrioventricular block (6%) (Table 1b). In-ICU, global mortality was 26%, and half of the deaths occurred within the first week of ICU admission (Additional file 2: Figure S2). The median length of stay was 6 (3–13) days in ICU and 19 (8–38) days in the hospital. Overall in-hospital mortality was 32%.

### Prognosis factors

**Fig. 3:**
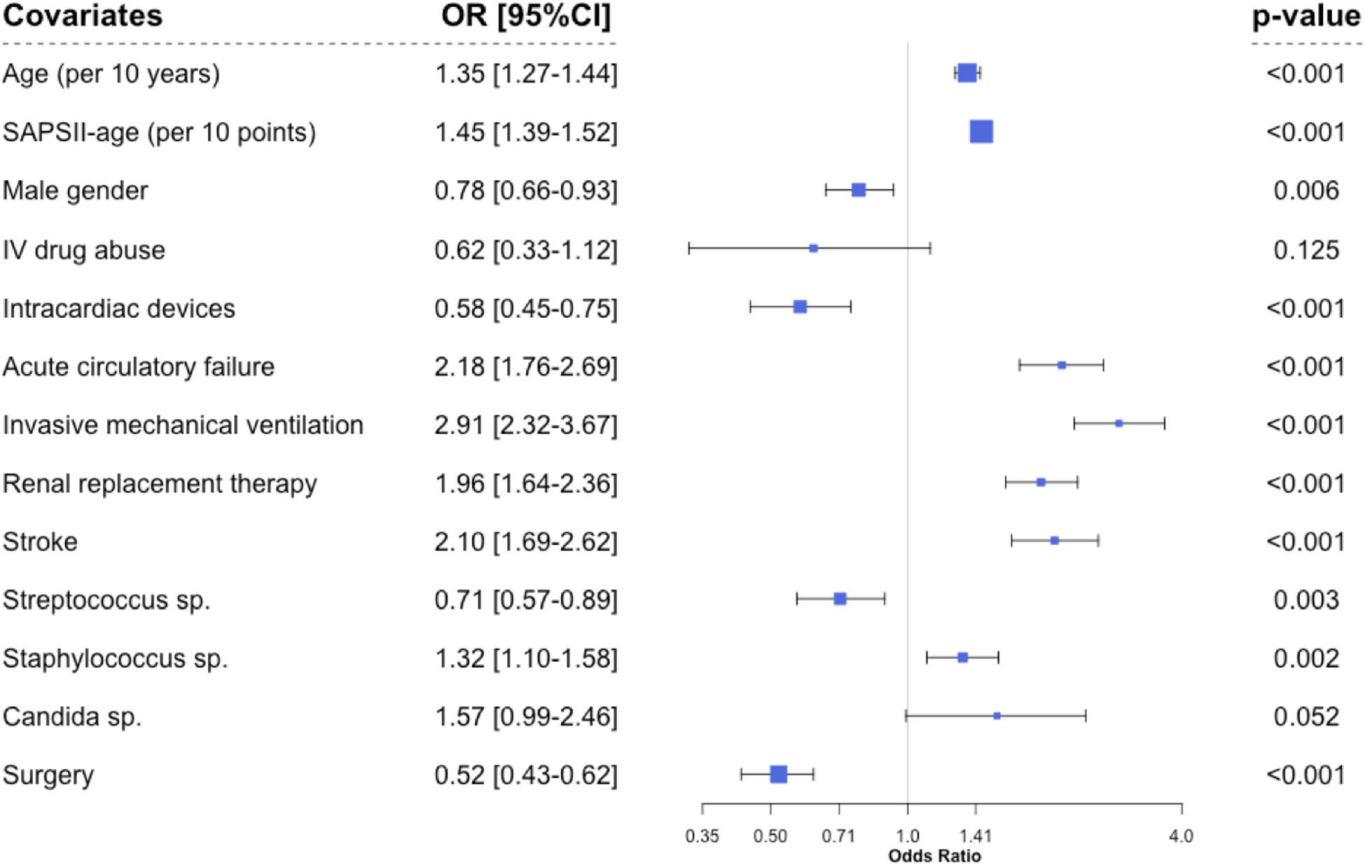
Multivariate analysis of risks factors for in-ICU mortality (logistic regression). Abbreviations: SAPS for Simplified Acute Physiology Score, RTT for renal replacement therapy, IV for intravenous. The dots represent the odds ratio; dot size is proportional to the odds ratio. The line through each dot corresponds to the 95% confidence interval. Variables with *P* < 0.10 entered in the maximal model for multivariate analysis. Goodness of fit (le Cessie-van Houwelingen statistic): *P* value = 0.13, calibration (AUC-ROC) 0.85.

We performed multivariate analysis on 4370 patients without missing data. By logistical regression, we identified several significant factors associated with in-ICU death (Additional file 3: Table S1A and B): age [OR 1.35 (1.27–1.44), *P* < 0.001], SAPS2 score minus age-related points [OR 1.45 (1.39–1.52), *P* < 0.001], male gender [OR 0.79 (0.66–0.93), *P* < 0.01], and intra-cardiac material [OR 0.58 (0.45–0.75)]. Organ failure was also associated with increased mortality, mainly due to acute respiratory failure requiring invasive mechanical ventilation [OR 2.91 (2.32–3.67), *P* < 0.001] and acute circulatory failure [OR 2.18 (1.76–2.69), *P* < 0.001]. Ischemic or hemorrhagic stroke and the need for RRT were also independently associated with mortality [OR 2.10 (1.69–2.62) and 1.96 (1.64–2.36), *P* < 0.001], respectively (Fig. 3). As regards to pathogens, *Staphylococcus* sp. was significantly associated with in-ICU death [OR 1.32 (1.10–1.58), *P* = 0.02], whereas *Streptococcus* sp. infection [OR 0.71 (0.57–0.89), *P* = 0.003] was associated with a lower risk of in-ICU mortality. Interestingly, we found a significant relationship between cardiac surgery for infective endocarditis and outcome [OR 0.52 (0.43–0.62), *P* < 0.0001] (Fig. 3). To better assess this association, we studied the effect of prognostic covariates in the subset of patients with and without surgery (Additional file 4: Figure S3). We did not find any significant interaction, underlying that surgical treatment was associated with in-ICU survival. These associations remained significant when the center was introduced as a random variable in the model (data not shown). Next, when the center was introduced as a cluster effect in the regression model, it was not associated with outcome (*P* = 0.10). We also studied the impact of prognostic covariates according to time-period, and thus, we found two significant interactions. As shown, the impact of circulatory failure and stroke on in-ICU mortality decreased over time (*P* = 0.04). In contrast, the effect of surgery on outcome seemed to increase along time, but this interaction was not significant (Additional file 5: Figure S4). Finally, we observed a global decrease in mortality over the study period (Additional file 6: Figure S5A) while patients’ severity increased. Therefore, the ratio between expected mortality and observed mortality predicted by the SAPS 2, representing an improvement in medical benefit, significantly increased over time (Additional file 6: Figure S5B).

### Interrupted time analysis over 1997–2003, 2004–2009, and 2010–2014

**Fig. 4:**
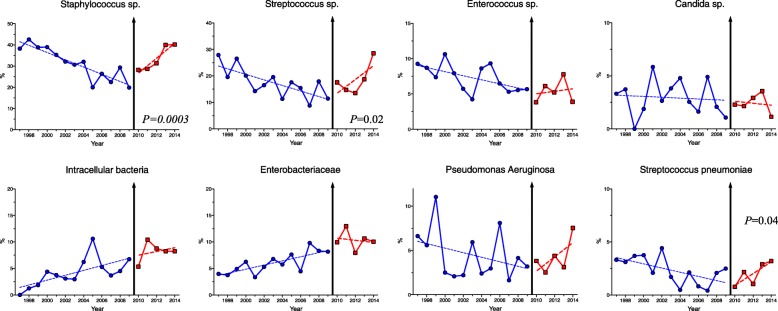
Culprit pathogens’ distribution over the time (expressed as percentage of infective endocarditis case) and interrupted time series analysis. Intracellular germs include *Coxiella Burnetii*, *Bartonella spp*., *Brucella spp*., *Chlamydia spp*., *Mycoplasma pneumoniae*, *Legionella pneumophila*, *Rickettsiae* sp., *Mycobacterium tuberculosis*, *and other Mycobacterium species*, *Francisella tularensis*, *Listeria monocytogenes*, *Nocardia spp.*

Period comparison highlighted profound changes in infective endocarditis epidemiology over the years (Additional file 7: Table S2): age (*P* < 0.0001) and severity (*P* < 0.0001) increased over the periods. Intra-cardiac material significantly increased (*P* < 0.0001), as well as surgery resort (from 27% during 1997–2003 to 42% in the 2010–2014). Endocarditis-related complications remained stable, except for high-grade atrioventricular block whose incidence dropped in the most recent period. Following American [14] and UK [15] guideline changes, *the European Society of Cardiology* has issued in 2009 new guidelines [16], arguing for a limitation of the indications for infective endocarditis antibiotic prophylaxis. To test whether these guidelines might have impacted on the microbial epidemiology of endocarditis patients admitted to ICU, we analyzed culprit microorganism’s proportion over the years. The distribution of culprit pathogens showed a significant change in slope for *Staphylococcus* sp. (*P* < 0.01), as well as for *Streptococcus* sp. (*P* = 0.03*)* with a U-shaped curve along time toward increased proportion of these pathogens in the most recent years (Fig. 4 and Additional file 7: Table S2), whereas previous trends slope remained unchanged for other microorganisms. Endocarditis due to *Staphylococcus* sp. infection is characterized by more frequent neurological complications, peripheral embolisms, and secondary septic localizations when compared to endocarditis due to other microorganisms (Additional file 8: Figure S6).

## Discussion

We described here the largest multicenter retrospective series focusing on critically ill infective endocarditis patients with organ failure requiring ICU admission in France over an 18-year period. A recent study in the USA over the same period [17] has been published with many similarities such as age, sex ratio, and prosthetic-valve endocarditis proportion. However, in our cohort, patients had more frequent organ failure and ultimately higher in-hospital mortality. Concerning causative microorganisms, *Streptococcus* sp. was less frequently involved in our series, while Gram-negative *bacilli* and *Candida* sp. were more frequently described. When compared to smaller size studies focusing on ICU population, the characteristics of our cohort (demographic data, mortality, and organ failure) were rather consistent [18, 19], but the use of valve surgery was lower. This difference could be explained because these studies were performed in tertiary care centers with cardiac surgery department and a higher proportion of prosthesis endocarditis [18–20]. One strength of our study is the low influence of referral bias because Cub-Rea database included patients from a large number of tertiary and primary care centers.

We observed an uninterrupted increase in the number of infective endocarditis cases in French ICUs, being 2-fold higher between 1997 and 2014, without any noticeable change in admission criteria or availabilities of ICU facilities over the periods. Duval et al. did not find any change of infective endocarditis incidence in French medical departments, but inclusions were stopped in 2008. Our observation is consistent with results from several groups in Europe who reported an increase of endocarditis incidence in the UK [5], in Denmark [21], in the Netherlands [22], and also in Germany [23]. Several hypotheses could be proposed to explain the increasing incidence over time. As life expectancy increases, people are exposed for a more extended period to predisposing factors such as degenerative valvular lesions, diabetes, cancer, and immunosuppressive drugs. In addition, more patients have prosthetic valves, intra-cardiac electronic devices, or long-term intravenous lines [24, 25]. In our cohort, age and cardiac material-related infective endocarditis significantly increased over time. In our study, mortality was 2-fold higher than another French cohort that included patients with less severe disease [26]. Nevertheless, mortality decreased over time, whereas SAPS2 [13] increased. Several factors could explain the improvement of prognosis over the years. In our study, we found the more frequent use of surgery as a factor associated with a better outcome. In a complementary statistical analysis, we did not find any heterogeneity in the effect of prognosis covariates in the subgroup of patients with and without surgery. This result suggests that surgery is an independent predictor of mortality independently of other covariates. This effect seemed to increase along time without significant association. This could reflect the improvement of the surgical procedure and the patient’s selection during the more recent periods. Recently, in a large Spanish population-based study including infective endocarditis patients from 2003 to 2014, Olmos et al. have reported during the same period a reduction of mortality and an increase of surgical interventions over time [6]. However, it is not possible to make a direct causative link between both epidemiological observations. We speculate that, besides surgical and anesthetic procedures improvement [27], patients have benefited from recent advances in the management of lung injury and acute circulatory failure in ICUs [28, 29]. Improvement in organ failure management in ICU could explain why we observed that acute circulatory failure impact on mortality decreased along time.

Over the years, surgery treatment increased. This finding is consistent with the recent modifications of IE surgical indications in international guidelines that recommend “emergency” or “urgent” valve surgery in cases of organ failure [30, 31]. The increasing prevalence of intra-cardiac material might also account for the higher necessity of surgery [32]. As we did not have exhaustive information concerning indications and time between diagnosis and surgical treatment, our study cannot contribute to clarify the debate about the effect of early versus delayed surgery [33] in patients with complicated infective endocarditis. Previous studies have reported that almost 75% of infective endocarditis patients in ICUs have an indication for surgery, but 50% of them have a contraindication because of multiple organ failure, poor general condition, or intracranial bleeding [19]. We cannot assess in our study the proportion of patients eligible for surgery but finally rejected. We included ischemic and hemorrhagic stroke in the multivariate analysis, and we observed that surgery still provided benefits, confirming previous studies [34].

We found that *Staphylococcus* sp. represented the more frequent causative pathogen family and correlate with poor prognosis. This observation confirmed previous studies in Europe [35] and the USA [4]. We observed in *Staphylococcus*-related endocarditis patients more frequent neurological complications, extra-cerebral embolisms, and secondary infectious localizations, compared to other pathogens. Otherwise, we observed that female gender was associated with a significantly higher risk of mortality. Our results are in line with Dohmen’s study that reported increased mortality in women with infective aortic endocarditis undergoing surgical treatment [36].

In parallel, we observed a changing landscape in causative microorganisms with an increase in *Staphylococcu*s and *Streptococcus* species. Several factors could account for these epidemiological observations, including aging and increased comorbidities among ICU patients, higher prevalence of intra-cardiac material [37], and improvement of infective endocarditis diagnosis methods. Based on our data, it is difficult to link the recent changes in antibioprophylaxis guidelines and the observed increased infective endocarditis incidence, specifically for the recent rebound in *Streptococcus sp.*-related endocarditis. Controversial studies on the impact of changes in antibioprophylaxis indication have been published [5, 38], and the design of our study is not fitted to address this issue.

### Limitations

The retrospective design of the present study using CUR-Rea database led to several limitations and potential bias. These issues, also observed in many large studies, are related to the complexity of the disease, at the diagnostic, and the therapeutic level. First of all, the diagnostic criteria for infectious endocarditis have changed over time, and from 2000, revised Duke’s criteria [39] replaced the criteria established by Durack in 1994 [40], effective at the beginning of our study. However, we believe that these minor changes do not induce a meaningful classification bias. To limit coding and diagnosis bias, we started data collection in 1997, when ICD-10 was introduced in France. In order to address the changes of coding practices of diagnoses over time, we assessed the coding of pulmonary embolism as a control and found that it did not significantly change throughout our study, suggesting a low bias related to coding (data not shown). In parallel, we performed internal quality control of our database on 97 medical charts in our center. Based on modified Duke s’ classification, we identified 86 definitive infective endocarditis, ten possible infective endocarditis, and only one rejected. Secondly, the ICD-10 diagnostic code for infectious endocarditis does not specify the valve(s) damages by itself. Also, we only have the valve involved in 1954 cases (44%), captured by the codes related to surgical procedures, which does not allow us to properly analyze the prognostic value of different locations. Thirdly, the features of ICD-10 pathogen-associated codes do not allow a detailed analysis of bacterial ecology. For example, many codes used for staphylococcal infections do not formally specify the culprit species or subspecies, and among S*treptococcaceae*, specific ICD-10 codes do not exist for oral Streptococci which would be fundamental to review the effect of guideline changes about antibiotic prophylaxis for dental procedures. At last, we have no available information about antibiotic therapy received by patients.

## Conclusion

This large multicenter study provides a unique overview of critically ill patients hospitalized for infective endocarditis and highlights a shifting landscape of epidemiology in French ICUs, characterized by improved prognosis despite higher patient severity, more surgery, and substantial changes in microbial epidemiology.

### Additional files

Additional file 1: **Figure S1.** Infective endocarditis case inclusion algorithm. (PPTX 63 kb)

Additional file 2: **Figure S2.** Estimated cumulative incidence of mortality as competing risk with being discharged alive from ICU. (PPTX 85 kb)

Additional file 3: **Table S1.** Characteristics of included infective endocarditis patients according to the outcome. (PPTX 47 kb)

Additional file 4: **Figure S3.** Study of covariates’ effect on ICU mortality according to surgery status. Interactions were tested by Gail and Simon LR test. The dots represent the odds ratio, and the line through each dot corresponds to the 95% confidence interval. *P* value < 0.05 is considered as significant. (PPTX 111 kb)

Additional file 5: **Figure S4.** Study of covariates’ effect on ICU mortality according to time period. Interactions were tested by Gail and Simon LR test. The dots represent the odds ratio, and the line through each dot corresponds to the 95% confidence interval. *P* value < 0.05 is considered as significant. (PPTX 119 kb)

Additional file 6: **Figure S5.** In-ICU observed mortality (A) and observed/expected mortality ratio (B). (PPTX 111 kb)

Additional file 7: **Table S2.** Comparison between 1997 and 2003, 2004–2009, and 2010–2014 periods. Abbreviations: MV, mechanical ventilation. (PPTX 235 kb)

Additional file 8: **Figure S6.** Infective endocarditis complications prevalence comparing *Staphylococcus* sp. vs. other pathogens. (PPTX 91 kb)

## Appendix

**Table 2:** Members of CUB-Rea database and email

Hospital department	Name
Hôpital Ambroise Paré	VIEILLARD-BARON Antoine
Hôpital André Mignot	BEDOS Jean-Pierre
Hôpital Antoine Béclère	TROUILLER Pierre
Hôpital Avicenne	COHEN Yves
Hôpital Bicêtre	RICHARD Christian
Hôpital Bichat	TIMSIT Jean françois
Hôpital Cochin	MIRA Jean-Paul
Hôpital Sud Francilien	CHEVREL Guillaume
Hôpital Delafontaine	DA SILVA Daniel
HEGP	DIEHL Jean-Luc
Hôpital de Gonesse	HO Paul
Hôpital Henri Mondor	MEKONTSO DESSAP Armand
Institut Gustave Roussy	BLOT François
Hôpital Lariboisière	MEGARBANE Bruno
Louis Mourier	DREYFUSS Didier
Montreuil	DAS Vincent
Hôpital Pitié (réa neuro)	BOLGERT Francis
Hôpital Pitié (pneumo)	DEMOULE Alexandre
Hôpital Pitié (chir-cardiaque)	COMBES Alain
Hôpital Poissy	OUTIN Hervé
CHI Le Raincy-Montfermeil	GOLDGRAN TOLEDANO Dany
Paul Brousse	SAMUEL Didier
Hôpital Raymond Poincaré	ANNANE Djillali
Hôpital Robert Ballanger	SANTOLI françois
Hôpital Saint Antoine	GUIDET Bertrand
Hôpital Saint Louis (Réa-med)	AZOULAY Elie
Hôpital Saint Louis (Réa-chir)	MEBAZAA Alexandre
Hôpital Saint-Joseph	BRUEL Cedric
Hôpital Tenon	BONNET Francis
Hôpital Tenon	FARTOUKH Muriel
Hôpital Victor Dupouy	MENTEC Hervé
Hôpital Jean Verdier	D’HONNEUR Gilles
