# Supplementary figures and images for: Epidemiology of infective endocarditis in French intensive care units over the 1997–2014 period—from CUB-Réa Network
Source: Crit Care. 2019 Apr 25;23:143. doi: 10.1186/s13054-019-2387-8 (PMC6485099; doi:10.1186/s13054-019-2387-8)

## Slide 1
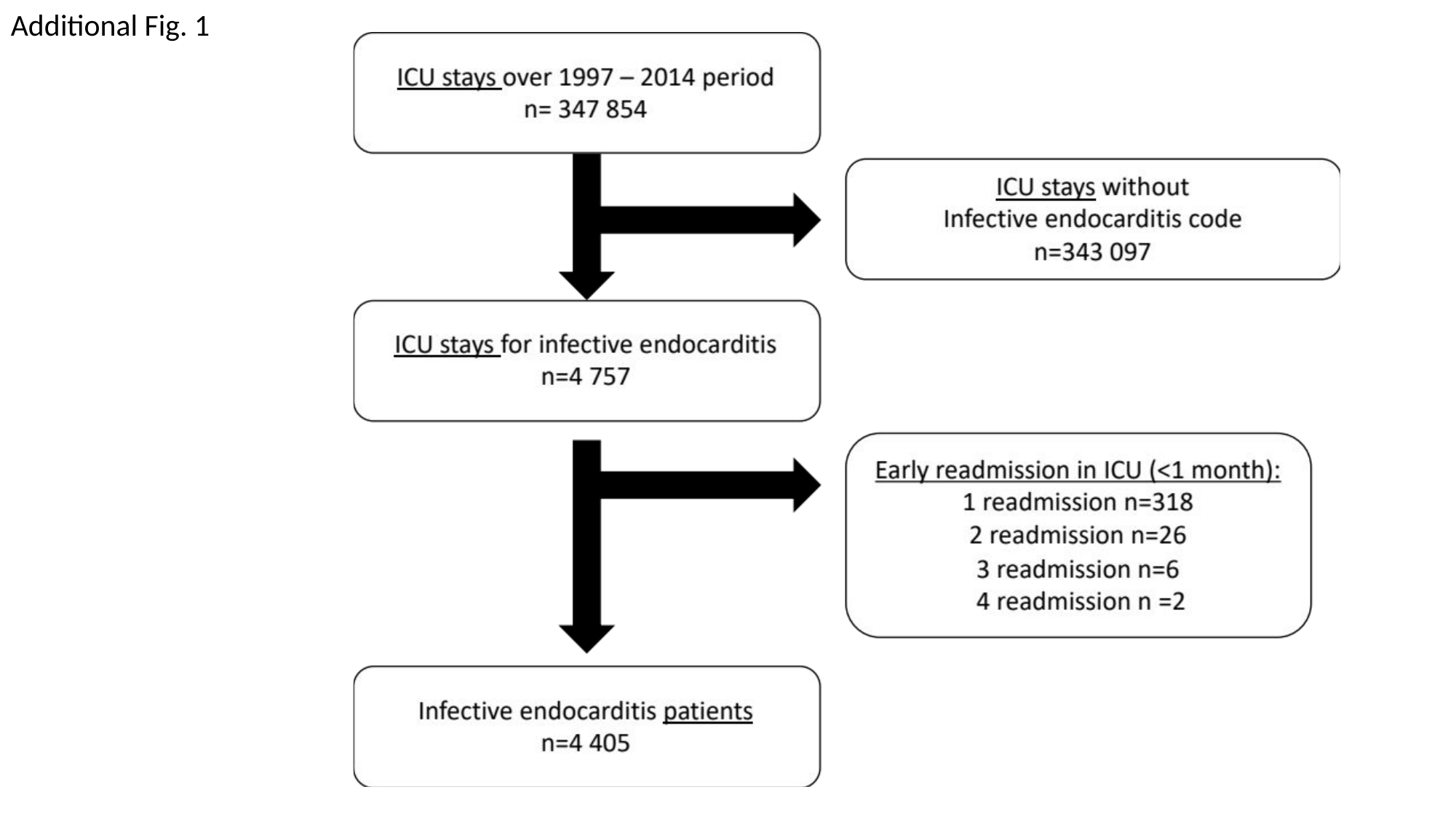

Additional Fig. 1

Supplement: Supplementary file 1 — Figure S1. Infective endocarditis case inclusion algorithm. (PPTX 63 kb) [file 13054_2019_2387_MOESM1_ESM.pptx]

## Slide 1
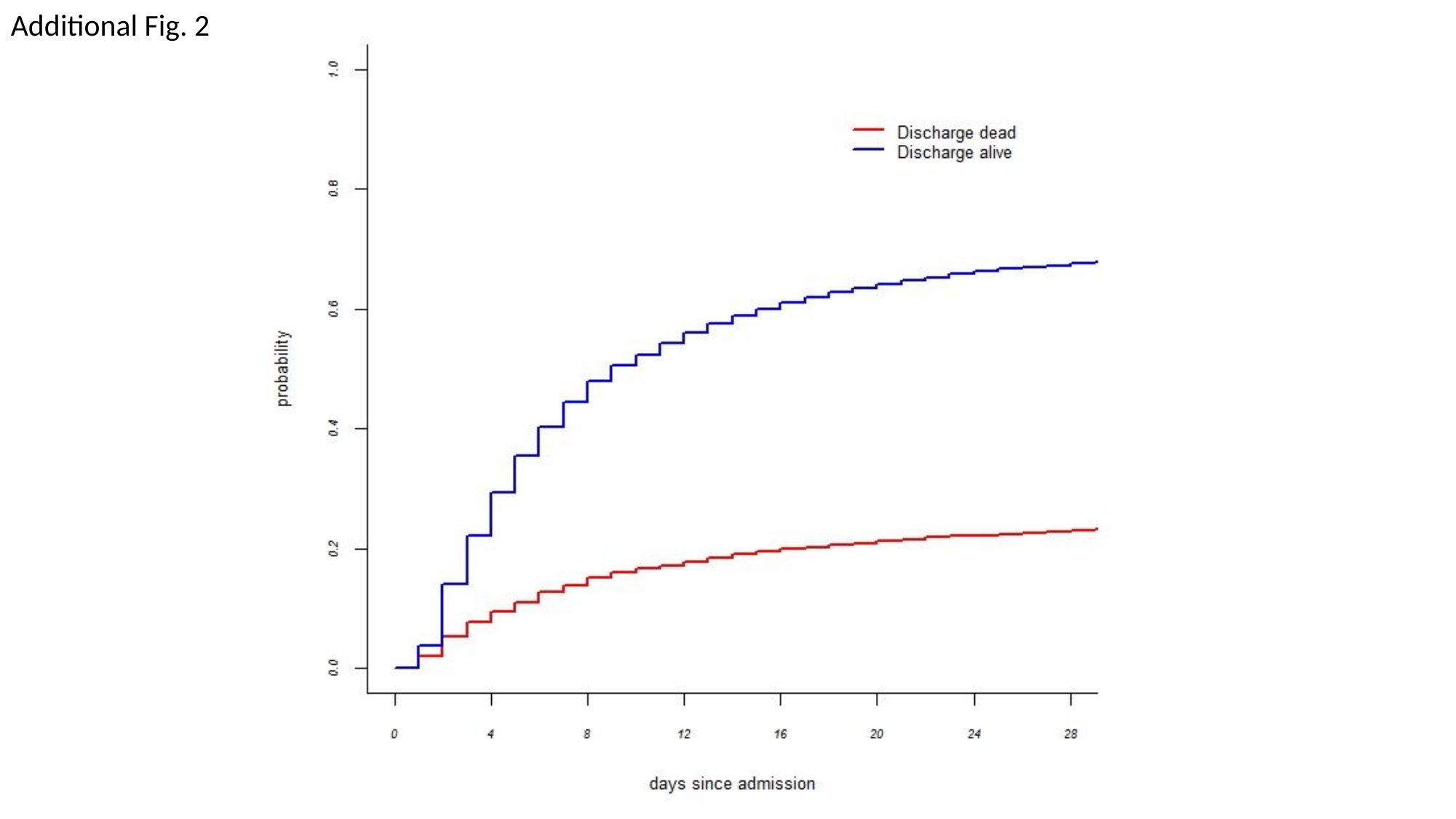

Additional Fig. 2

Supplement: Supplementary file 2 — Figure S2. Estimated cumulative incidence of mortality as competing risk with being discharged alive from ICU. (PPTX 85 kb) [file 13054_2019_2387_MOESM2_ESM.pptx]

## Slide 1
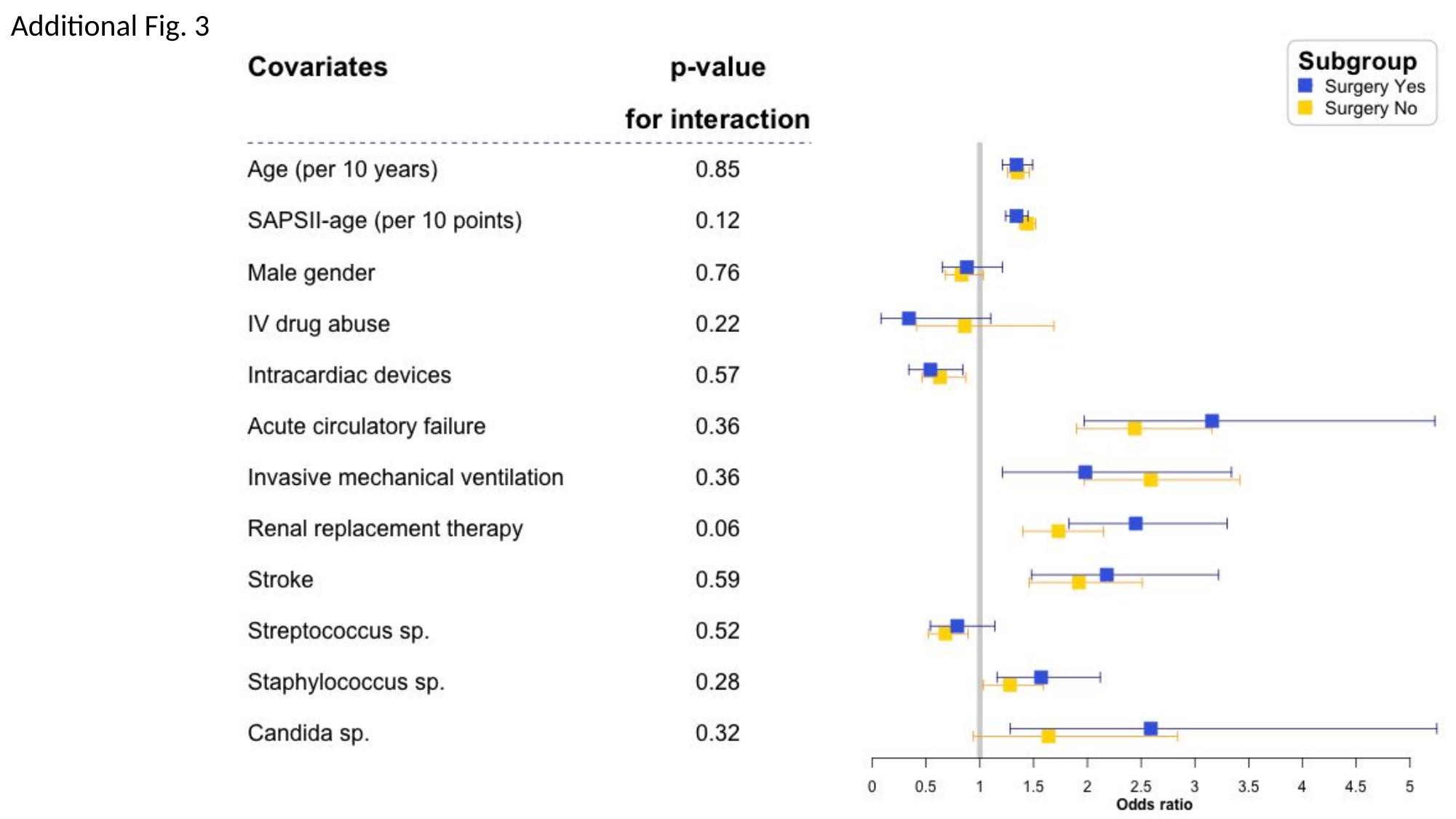

Additional Fig. 3

Supplement: Supplementary file 4 — Figure S3. Study of covariates’ effect on ICU mortality according to surgery status. Interactions were tested by Gail and Simon LR test. The dots represent the odds ratio, and the line through each dot corresponds to the 95% confidence interval. P value < 0.05 is considered as significant. (PPTX 111 kb) [file 13054_2019_2387_MOESM4_ESM.pptx]

## Slide 1
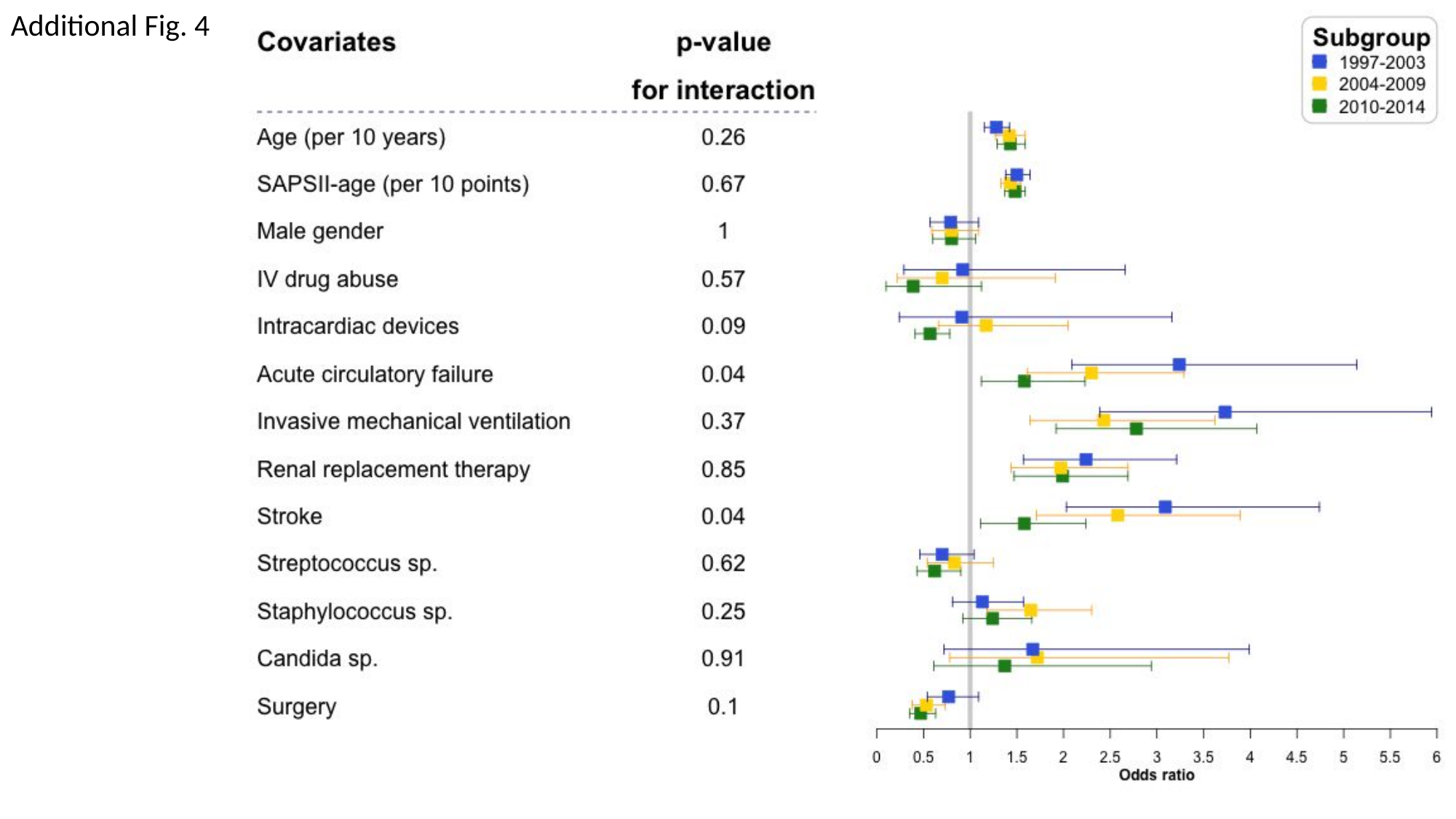

Additional Fig. 4

Supplement: Supplementary file 5 — Figure S4. Study of covariates’ effect on ICU mortality according to time period. Interactions were tested by Gail and Simon LR test. The dots represent the odds ratio, and the line through each dot corresponds to the 95% confidence interval. P value < 0.05 is considered as significant. (PPTX 119 kb) [file 13054_2019_2387_MOESM5_ESM.pptx]

## Slide 1
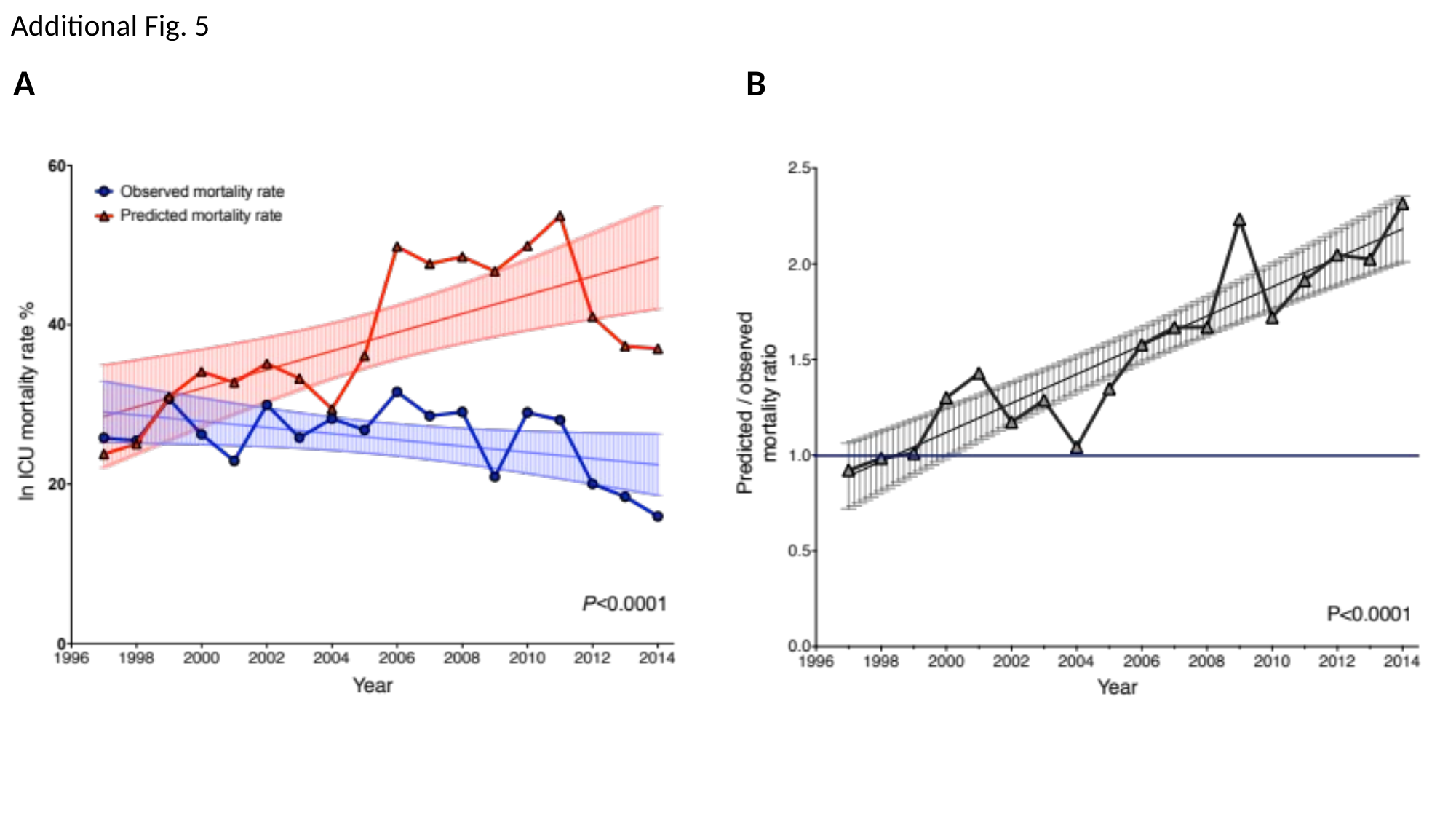

Additional Fig. 5
A
B

Supplement: Supplementary file 6 — Figure S5. In-ICU observed mortality (A) and observed/expected mortality ratio (B). (PPTX 111 kb) [file 13054_2019_2387_MOESM6_ESM.pptx]

## Slide 1
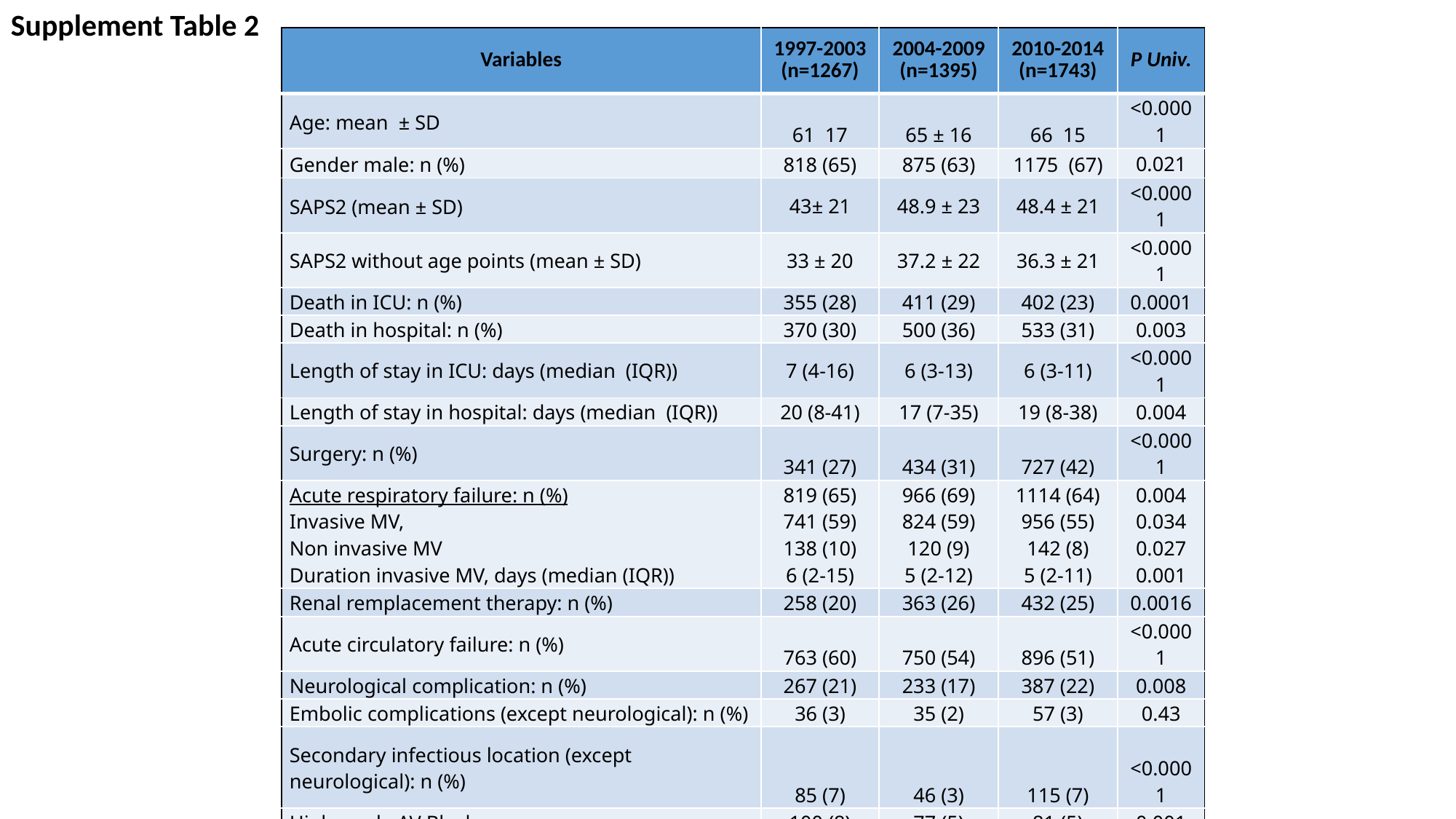

Supplement Table 2

Supplement: Supplementary file 7 — Table S2. Comparison between 1997 and 2003, 2004–2009, and 2010–2014 periods. Abbreviations: MV, mechanical ventilation. (PPTX 235 kb) [file 13054_2019_2387_MOESM7_ESM.pptx]

## Slide 1
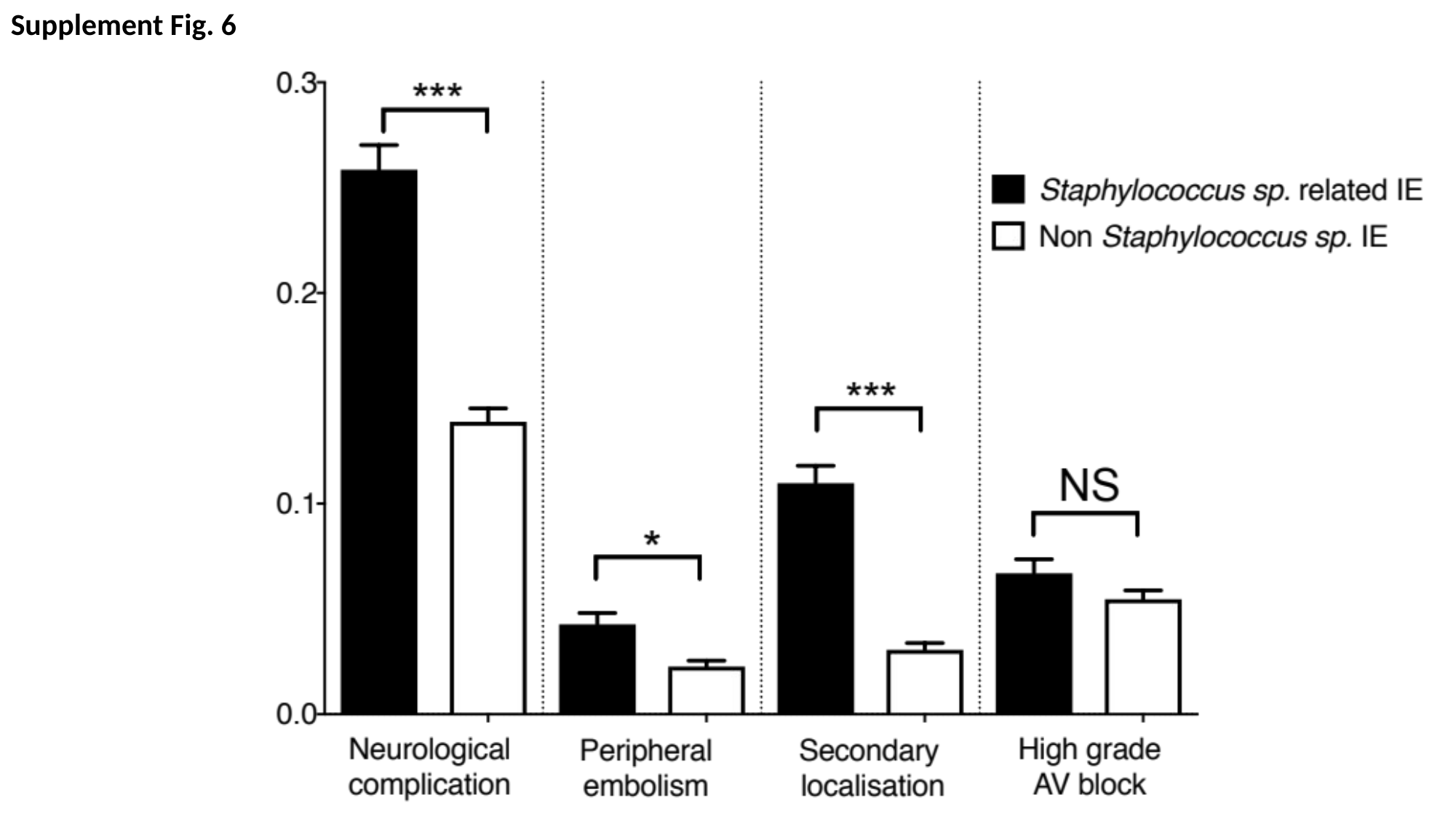

Supplement Fig. 6

Supplement: Supplementary file 8 — Figure S6. Infective endocarditis complications prevalence comparing Staphylococcus sp. vs. other pathogens. (PPTX 91 kb) [file 13054_2019_2387_MOESM8_ESM.pptx]
