# Supplementary material for: Epidemiology of infective endocarditis in French intensive care units over the 1997–2014 period—from CUB-Réa Network
Source: Crit Care. 2019 Apr 25;23:143. doi: 10.1186/s13054-019-2387-8 (PMC6485099; doi:10.1186/s13054-019-2387-8)
Supplement: Supplementary file 3 — Table S1. Characteristics of included infective endocarditis patients according to the outcome. (PPTX 47 kb) [file 13054_2019_2387_MOESM3_ESM.pptx]

## Slide 1
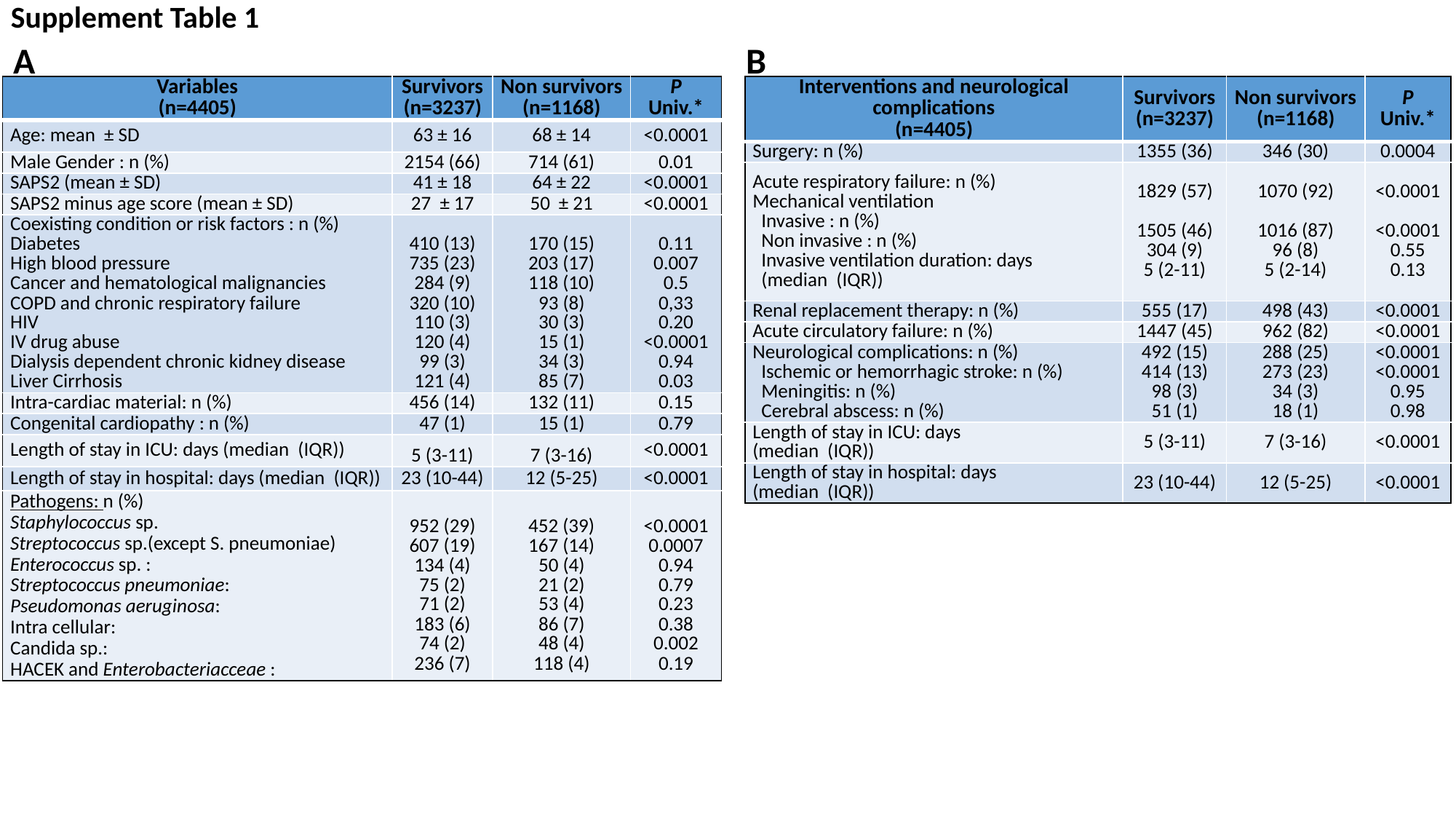

Supplement Table 1
A
B
| Variables (n=4405) | Survivors (n=3237) | Non survivors (n=1168) | P Univ.\* |
| --- | --- | --- | --- |
| Age: mean ± SD | 63 ± 16 | 68 ± 14 | <0.0001 |
| Male Gender : n (%) | 2154 (66) | 714 (61) | 0.01 |
| SAPS2 (mean ± SD) | 41 ± 18 | 64 ± 22 | <0.0001 |
| SAPS2 minus age score (mean ± SD) | 27 ± 17 | 50 ± 21 | <0.0001 |
| Coexisting condition or risk factors : n (%) Diabetes High blood pressure Cancer and hematological malignancies COPD and chronic respiratory failure HIV IV drug abuse Dialysis dependent chronic kidney disease Liver Cirrhosis | 410 (13) 735 (23) 284 (9) 320 (10) 110 (3) 120 (4) 99 (3) 121 (4) | 170 (15) 203 (17) 118 (10) 93 (8) 30 (3) 15 (1) 34 (3) 85 (7) | 0.11 0.007 0.5 0,33 0.20 <0.0001 0.94 0.03 |
| Intra-cardiac material: n (%) | 456 (14) | 132 (11) | 0.15 |
| Congenital cardiopathy : n (%) | 47 (1) | 15 (1) | 0.79 |
| Length of stay in ICU: days (median (IQR)) | 5 (3-11) | 7 (3-16) | <0.0001 |
| Length of stay in hospital: days (median (IQR)) | 23 (10-44) | 12 (5-25) | <0.0001 |
| Pathogens: n (%) Staphylococcus sp. Streptococcus sp.(except S. pneumoniae) Enterococcus sp. : Streptococcus pneumoniae: Pseudomonas aeruginosa: Intra cellular: Candida sp.: HACEK and Enterobacteriacceae : | 952 (29) 607 (19) 134 (4) 75 (2) 71 (2) 183 (6) 74 (2) 236 (7) | 452 (39) 167 (14) 50 (4) 21 (2) 53 (4) 86 (7) 48 (4) 118 (4) | <0.0001 0.0007 0.94 0.79 0.23 0.38 0.002 0.19 |
| Interventions and neurological complications (n=4405) | Survivors (n=3237) | Non survivors (n=1168) | P Univ.\* |
| --- | --- | --- | --- |
| Surgery: n (%) | 1355 (36) | 346 (30) | 0.0004 |
| Acute respiratory failure: n (%) Mechanical ventilation Invasive : n (%) Non invasive : n (%) Invasive ventilation duration: days (median (IQR)) | 1829 (57)   1505 (46) 304 (9) 5 (2-11) | 1070 (92)   1016 (87) 96 (8) 5 (2-14) | <0.0001   <0.0001 0.55 0.13 |
| Renal replacement therapy: n (%) | 555 (17) | 498 (43) | <0.0001 |
| Acute circulatory failure: n (%) | 1447 (45) | 962 (82) | <0.0001 |
| Neurological complications: n (%) Ischemic or hemorrhagic stroke: n (%) Meningitis: n (%) Cerebral abscess: n (%) | 492 (15) 414 (13) 98 (3) 51 (1) | 288 (25) 273 (23) 34 (3) 18 (1) | <0.0001 <0.0001 0.95 0.98 |
| Length of stay in ICU: days (median (IQR)) | 5 (3-11) | 7 (3-16) | <0.0001 |
| Length of stay in hospital: days (median (IQR)) | 23 (10-44) | 12 (5-25) | <0.0001 |
